# Classification of K-Pop Dance Movements Based on Skeleton Information Obtained by a Kinect Sensor

**DOI:** 10.3390/s17061261

**Published:** 2017-06-01

**Authors:** Dohyung Kim, Dong-Hyeon Kim, Keun-Chang Kwak

**Affiliations:** 1 Electronics and Telecommunications Research Institute (ETRI), Daejeon 34129, Korea; dhkim008@etri.re.kr; 2 Department of Control and Instrumentation Engineering, Chosun University, Gwangju 61452, Korea; aw981@daum.net

**Keywords:** dimensionality reduction, extreme learning machine, fisherdance, K-pop dance movements, skeletal joint data

## Abstract

This paper suggests a method of classifying Korean pop (K-pop) dances based on human skeletal motion data obtained from a Kinect sensor in a motion-capture studio environment. In order to accomplish this, we construct a K-pop dance database with a total of 800 dance-movement data points including 200 dance types produced by four professional dancers, from skeletal joint data obtained by a Kinect sensor. Our classification of movements consists of three main steps. First, we obtain six core angles representing important motion features from 25 markers in each frame. These angles are concatenated with feature vectors for all of the frames of each point dance. Then, a dimensionality reduction is performed with a combination of principal component analysis and Fisher’s linear discriminant analysis, which is called fisherdance. Finally, we design an efficient Rectified Linear Unit (ReLU)-based Extreme Learning Machine Classifier (ELMC) with an input layer composed of these feature vectors transformed by fisherdance. In contrast to conventional neural networks, the presented classifier achieves a rapid processing time without implementing weight learning. The results of experiments conducted on the constructed K-pop dance database reveal that the proposed method demonstrates a better classification performance than those of conventional methods such as KNN (K-Nearest Neighbor), SVM (Support Vector Machine), and ELM alone.

## 1. Introduction

The past decade has witnessed rapid growth in the number of motion capture applications, ranging from sports sciences and motion analysis to motion-based video games and movies [1,2,3,4,5]. Generally defined, motion capture is the process of recording the movements of humans. It refers to recording the actions of human actors and using that information to animate digital character models in 2D or 3D computer animation sequences. Recently, we have also witnessed the popularity of Korean pop (K-pop) music spread throughout the world. K-pop is a musical genre originating from South Korea that is characterized by a wide variety of audiovisual elements. Although it includes all genres of popular music in South Korea, the term is more often used in a narrower sense to describe a modern form of South Korean pop music covering a range of styles including dance-pop, pop ballads, electro-pop, rock, jazz, and hip-pop. One possible reason that K-pop has become so popular globally is that other aspiring dancers may feel inclined to view skilled young K-pop dancers as role models and to copy their dance styles. This can lead to plagiarism issues in both dance and music, which is our main motivation for classifying K-pop dance movements for the development of both video-based retrieval systems and dance training systems.

There are three main types of motion capture systems: optical systems, non-optical systems, and markerless systems. Optical systems use the data captured from optical sensors to detect the 3D positions of a subject located between two or more cameras that are calibrated to provide overlapping projections. Data acquisition is traditionally implemented by attaching special markers to the actor. Optical capture systems are used with several types of markers, including passive markers, active markers, time modulated active markers, and semi-passive imperceptible markers. Non-optical capture systems include inertial systems, mechanical motion systems, and magnetic systems. Among these, inertial motion capture is the best-known capture system. Inertial motion capture technology includes inertial sensors, biomechanical models, and sensor fusion algorithms. Inertial motion-sensor data are often transmitted wirelessly to a computer, where the motion is recorded or viewed. Finally, the markerless capture method is currently assisting the rapid development of the markerless approach to motion capture in the area of computer vision. Markerless systems do not require subjects to wear special equipment for tracking. Several studies related to markerless systems have been performed via motion analysis of data obtained from the well-known Kinect sensor [6,7,8,9,10,11,12,13,14,15].

In this paper, we focus on a markerless capture method based on the skeletal joint data of human motion utilizing a Kinect camera in a motion-capture studio environment for the classification of K-pop dance movements. The previous works have been focused on ballet analysis [16,17], video recommendation based on dance styles [18], dance pose estimation [19,20], dance animation [21], and e-learning of dance [22]. While some ballet movements and dance pose estimation have previously been studied in various aspects [16,17,18,19,20,21,22,23,24,25,26], nobody has yet performed research on K-pop dance movements using Kinect sensors to address the problem of dance plagiarism. In order to accomplish this, a K-pop dance database is constructed from the motions of professional dancers. The process of dance movement classification comprises feature extraction, dimensionality reduction, and, finally, the classification itself. In the first step, features are extracted from 25 markers of skeletal joint data. We use six features representing the important motion angles in each frame. These features are connected in the form of a feature vector for all of the frames. Next, a combination of principal component analysis (PCA) [27] and linear discriminant analysis (LDA) [28], referred to in this paper as “fisherdance”, is performed to reduce the dimensionality of the dance movements. In the last step, an extreme learning machine classifier (ELMC) is designed based on a rectified linear unit (ReLU)-based activation function. The characteristics of the ReLU-based ELMC are high accuracy, low user intervention, and real-time learning that occurs in seconds or milliseconds. Conventional ELMs have homogenous architectures for compression, feature learning, clustering, regression, and classification. Research has been conducted on the use of ELMs in various applications, including image super-resolution [29], real operation of wind farms [30], electricity price forecasting [31], remote control of a robotic hand [32], human action recognition [33], and 3D shape segmentation and labeling [34]. A considerable number of studies have been conducted on ELM variants [35,36,37,38,39,40]. The results of experiments performed on the constructed database demonstrate that the classification performance of the proposed method outperforms those employed in these studies.

This paper is organized in the following manner. Section 2 describes the generation of the concatenated vectors from the six core angles of each frame as well as the dimensionality reduction method utilized in this study. Section 3 describes the techniques used in dance movement classification realized via the ReLU-ELMC. Section 4 covers the results of simulations performed on the K-pop dance databases available at the Electronics and Telecommunications Research Institute (ETRI). Finally, Section 5 includes our concluding comments. 

## 2. Dimensionality Reduction of Concatenated Vectors

In this section, we describe a dimensionality reduction method using both PCA and LDA. The dimensional reduction exploited here consists of a three-phase development process. First, concatenated vectors are produced from six important angles specifying K-pop dance movements. Next, the PCA is performed by projecting the high-dimensional vectors into lower-dimensional spaces. Finally, feature vectors with discriminating capabilities are obtained by the LDA.

### 2.1. Generating Concatenated Vectors

**Figure 1:**
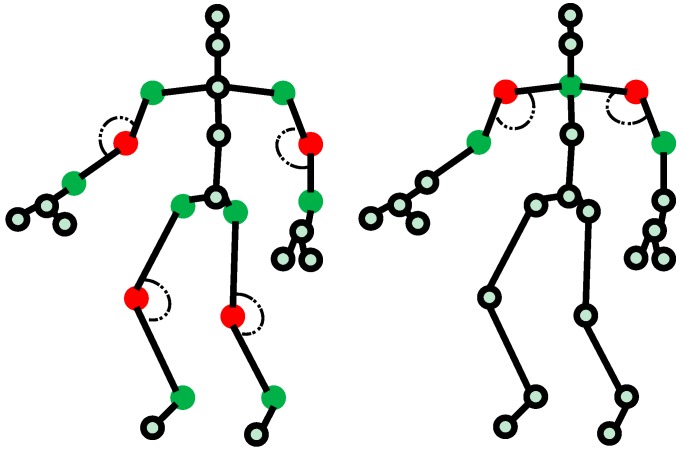
Six core angles distinguishing each dance movement.

**Figure 2:**
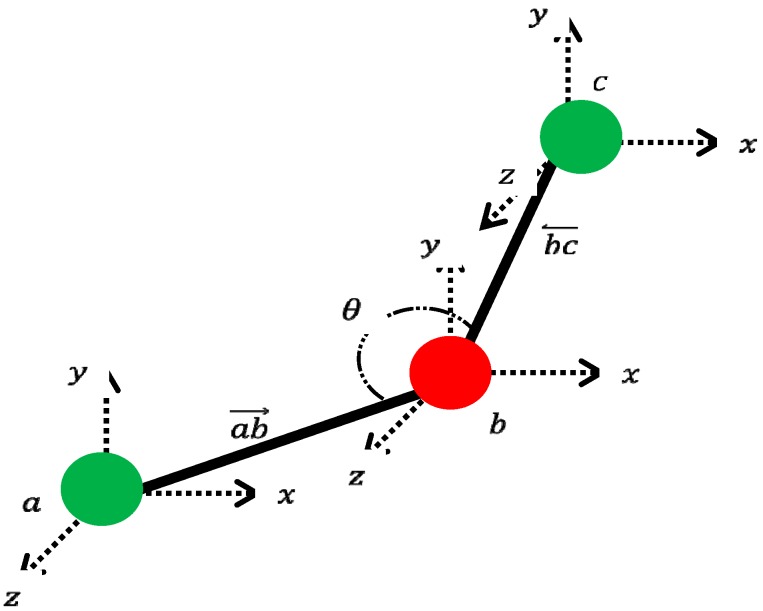
Angle between two neighboring joints.

In the first stage of our analysis, concatenated vectors are generated. Figure 1 illustrates the six core angles that distinguish each dance movement. As shown in Figure 1, these angles are related to the positions of both elbows, both knees, and both shoulders. Figure 2 illustrates an angle between two joints. This angle is calculated with the following equations:

(1) $$\vec{ab}=(x_{a}-x_{b},y_{a}-y_{b},z_{a}-z_{b})$$

(2) $$\vec{bc}=(x_{c}-x_{b},y_{c}-y_{b},z_{c}-z_{b})$$

(3) $$\theta =\cos^{-1}\frac{\vec{ab}\cdot \vec{bc}}{\left|\vec{ab}\right|\cdot \left|\vec{bc}\right|}$$

**Figure 3:**
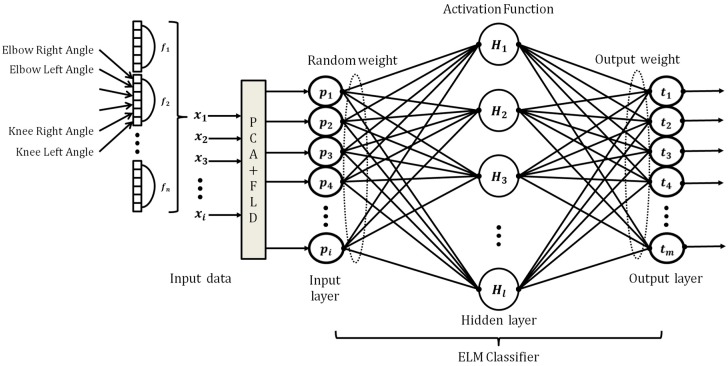
Architecture of the proposed method.

The total concatenated angles are generated by connecting these values within each frame, as shown in Figure 3. In general, the frame lengths of dance movements differ according to the dance type. To solve this problem, we perform a zero-padding method to set the frame sizes to the same size as the largest frame. For example, if the number of frames in a certain dance movement is 200, the size of the concatenated vector for each dance movement is 6 × 200 frames.

### 2.2. Combination of PCA and LDA for Dimensional Reduction

The method combining PCA and LDA for dimensional reduction is insensitive to large variations in movement. By maximizing the ratio of the between-scatter matrix to the within-scatter matrix, LDA produces well-separated dance movement categories in a low-dimensional subspace. In what follows, we briefly describe the method referred to as “fisherdance” in this work as the well-known fisherface method [19]. This method consists of the two steps shown in Figure 3. In the first step, the PCA projects the concatenated vectors from a high-dimensional image space into a lower-dimensional space. In the second step, the LDA finds the optimal projection from a classification perspective, which is known as a class-specific method. Therefore, we can perform this step by first projecting the K-pop dance movement into a lower-dimensional space using the combination of PCA and LDA, so that the resulting within-class scatter matrix is nonsingular, before computing the optimal projection. 

We denote the training set of N different dance movements as $Z=(z_{1},z_{2},\ldots ,z_{N})$ and define the covariance matrix as follows:

(4) $$R=\frac{1}{N}\sum_{i=1}^{N}(z_{i}-\bar{z}){\left(z_{i}-\bar{z}\right)}^{T}=\Phi \Phi^{T},$$

(5) $$\bar{z}=\frac{1}{N}\sum_{i=1}^{N}z_{i},$$

where $z_{i}$ is the concatenated vector of a dance movement. Then, both the eigenvalues and eigenvectors of the covariance matrix R are calculated. Let $E=(e_{1},e_{2},\cdots ,e_{r})$ contain the eigenvectors corresponding to the largest eigenvalues. For a set of original dance movements Z, the corresponding reduced feature vectors, $X=(x_{1},x_{2},\ldots ,x_{N})$, can be obtained by projecting Z into the PCA-transformed space according to the following equation:

(6) $$x_{i}=E^{T}(z_{i}-\bar{z}).$$

The second step, which is based on the use of the LDA, can be described as follows. Consider *c* classes with *N* samples each. Let the between-class scatter matrix be defined as

(7) $$S_{B}=\sum_{i=1}^{c}N_{i}(m_{i}-\bar{m})(m_{i}-\bar{m}{)}^{T},$$

where $N_{i}$ is the number of samples in the *i*th class $C_{i}$, $\bar{m}$ is the mean of all of the samples, and $m_{i}$ is the mean of class $C_{i}$. The within-class scatter matrix is defined as

(8) $$S_{W}=\sum_{i=1}^{c}\sum_{x_{k}\in C_{i}}^{}(x_{k}-m_{i})(x_{k}-m_{i}{)}^{T}=\sum_{i=1}^{c}S_{W_{i}},$$

where $S_{W_{i}}$ is the covariance matrix of class $C_{i}$. The optimal projection matrix, $W_{FLD}$, is obtained as the matrix with orthonormal columns that maximize the ratio of the determinant of the projected samples’ between-class matrix to their determinant of the within-class scatter matrix, as in the following expression:

(9) $$W_{FLD}=\arg\underset{W}{\max}\frac{\left|W^{T}S_{B}W\right|}{\left|W^{T}S_{W}W\right|}=\left[\begin{array}{cccc} w_{1} & w_{2} & \cdots & w_{m} \end{array}\right],$$

where $\left\{w_{i}|i=1,2,\cdots ,m\right\}$ is the set of generalized discriminant vectors of both $S_{B}$ and $S_{W}$ corresponding to the c−1 largest generalized eigenvalues $\left\{\lambda_{i}|i=1,2,\cdots ,m\right\}$, i.e.,

(10) $$S_{B}w_{i}=\lambda_{i}S_{W}w_{i}\;i=1,2,\ldots ,m.$$

Thus, the feature vectors $V=(v_{1},v_{2},\ldots ,v_{N})$ for any dance movement $z_{i}$ can be calculated as follows:

(11) $$v_{i}=W_{FLD}^{T}x_{i}=W_{FLD}^{T}E^{T}(z_{i}-\bar{z}).$$

To complete the classification of a new dance pattern $z^{\prime }$, we compute the distance between $z^{\prime }$ and a pattern in the training set z such that

(12) $$d(z,z^{\prime })=\left\|v-v^{\prime }\right\|.$$

The measure $d(z,z^{\prime })$ is defined as the distance between the training dance movement z and a given movement $z^{\prime }$ in the test set. Note that this distance is computed based on both v and $v^{\prime }$, which are the LDA-transformed feature vectors of dance movements z and $z^{\prime }$, respectively. While the distance function ‖⋅‖ can be broadly interpreted, quite often we confine ourselves to the Euclidean distance.

## 3. Design of ReLU-Based ELMC

In this section, we design the ReLU-based ELMC based on the feature vectors obtained by the PCA and LDA. This classifier possesses the important characteristics of both a simple tuning-free network and a fast learning speed. Unlike those in conventional existence theories, the node parameters hidden in the design of an ELM are independent of the training data. Although hidden nodes are both important and critical, these nodes generally do not need to be tuned. 

### ELMC

Most studies on neural networks are performed based on conventional existence theories, including those of the adjustment and learning of hidden nodes. Many researchers have performed intensive research on developing good learning methods over the past few decades. In contrast to conventional neural networks, we develop an ELMC with real-time learning and high classification abilities for classifying dance movements. Figure 3 shows the architecture of the ELMC. Given random hidden neurons that need not be either algebraic sums or other ELM feature mappings, almost all nonlinear piecewise continuous hidden nodes can be represented as follows:

(13) $$H_{i}(x)=G_{i}(a_{i},b_{i},x),$$

where $a_{i}$ and $b_{i}$ are the weight and the bias between the input and hidden layers, respectively. Although we do not know true output functions of biological neurons, most of them are nonlinear piecewise continuous functions covered by ELM theories. The output function of a generalized single layer feedforward network is expressed as

(14) $$f_{L}(x)=\sum_{i=1}^{L}\beta_{i}G_{i}(a_{i},b_{i},x).$$

The output function of the hidden layer mapping is as follows:

(15) $$H(x)=\left[G_{1}(a_{1},b_{1},x),\;\cdots ,\;G_{L}(a_{L},b_{L},x)\right].$$

The output functions of hidden nodes can be used in various forms. Many different types of learning algorithms exist, including sigmoid networks, radial basis function (RBF) networks, polynomial networks, complex networks, Fourier series networks, and wavelet networks, some of which are represented by:

$$\text{Sigmoid}:\;G(a_{i},b_{i},x)=g(a_{i}\cdot x+b_{i})\;\text{RBF}:\;G(a_{i},b_{i},x)=g(b_{i}\left\|x-a_{i}\right\|)\;\text{Fourier series}:\;G(a_{i},b_{i},x)=\cos(a_{i}\cdot x+b_{i})\;\text{Random projection}:\;G(a_{i},b_{i},x)=a_{i}\cdot x$$

where conventional random projection is just a specific case of ELM random feature mapping when an additive linear hidden node is used. This not only proves the existence of the networks but also provides learning solutions. In this paper, we use the ReLU-based activation function that is utilized effectively in convolutional neural networks and is given as follows:

(16) f(x)=max(0,x),

where **x** is the input to a neuron. In contrast to the sigmoid function, the major advantage of the ReLU function is in solving the vanishing gradient problem in neural network design. Furthermore, the constant ReLU function gradient results in faster learning. 

Given a training set $\left\{\left(x_{i},t_{i}\right)|x_{i}\in R^{d},t_{i}\in R^{m},\;i=1,2,\;\cdots ,N\right\}$, the hidden node output function G(a,b,x), and the number of hidden nodes L, the ELM determines both the hidden node parameters and the output weights using the following three-steps:

[Step 1] Assign the hidden node parameters randomly $(a_{i},b_{i}),\;i=1,2,\cdots ,N$

[Step 2] Calculate the hidden layer output matrix $H=\left[\begin{array}{c} h(x_{1})\\ \vdots \\ h(x_{N}) \end{array}\right]$

[Step 3] Calculate the output weights β using the least square estimate with

(17) $$\beta ={Η}^{†}T,$$

where $H^{†}$ is the Moore-Penrose generalized inverse of matrix H. When $H^{T}H$ is nonsingular, $H^{†}={\left(H^{T}H\right)}^{-1}H^{T}$. The significant features of ELM are summarized in the following.

First, the hidden layer does not need to be tuned. Second, the hidden layer mapping h(x) satisfies universal approximation conditions. Third, the parameters of ELM are minimized as follows:

(18) $${\left\|H\beta -T\right\|}_{p}.$$

ELM satisfies both the ridge regression theory and the neural network generalization theory. Finally, it fills the gaps and builds bridges among neural networks, SVMs, random projections, Fourier series, matrix theories, and linear systems.

**Figure 4:**
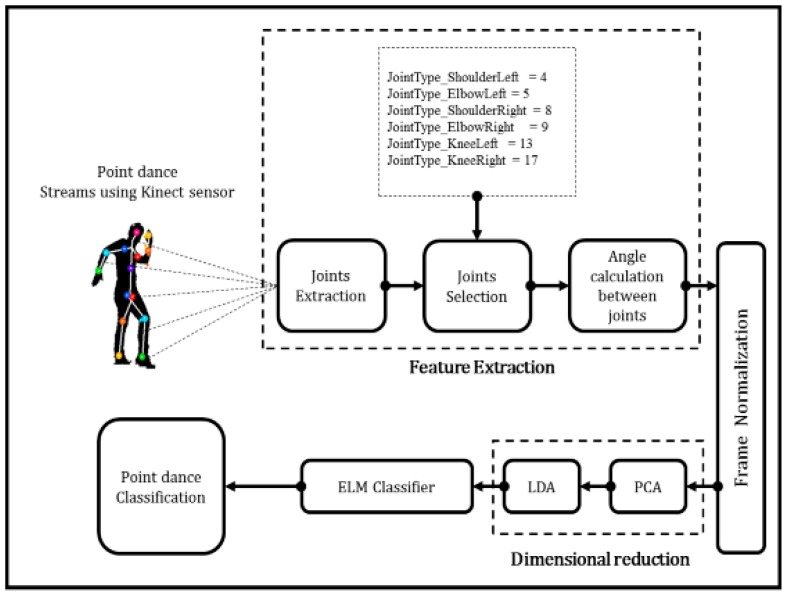
Point dance classification process flow.

Figure 4 shows the point-dance classification process flow regarding angle calculation between joints, frame normalization, dimensional reduction, and ELM classifiers. 

## 4. Experimental Results

This section reports on a comprehensive set of comparative experiments performed to evaluate the performance of the proposed approach.

### 4.1. Construction of K-Pop Dance Database

**Figure 5:**
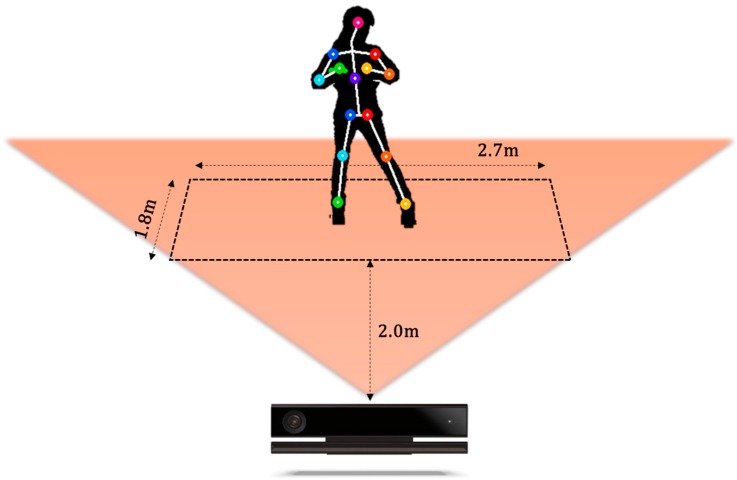
Database construction environment.

**Figure 6:**
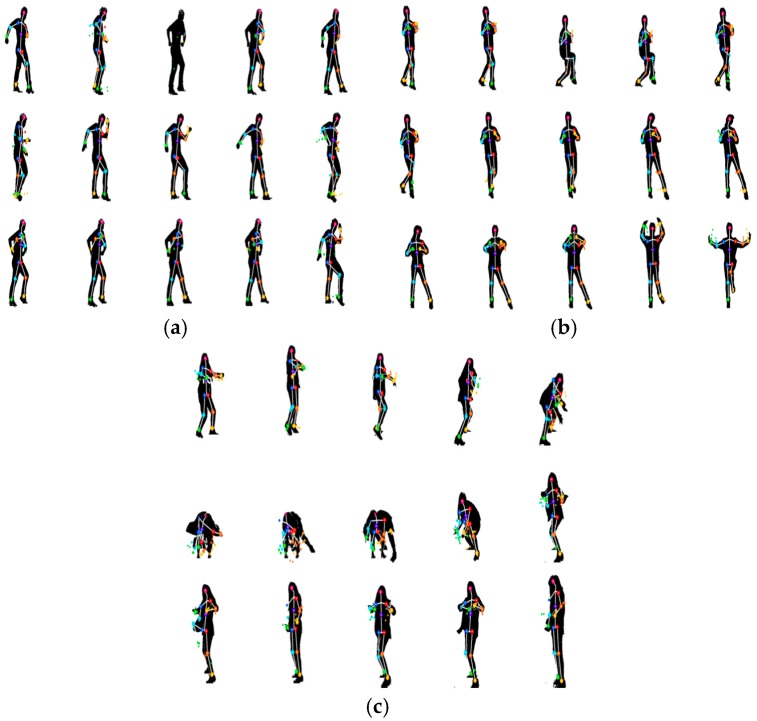
Three examples of dance movements (**a**) dance 1; (**b**) dance 2; (**c**) dance 3.

A K-pop dance database was constructed containing 200 point-dance movements from four professional dancers (two men and two women) obtained by a motion capture system that produced skeletal forms. Thus, there were 800 dance-movement data points in total. In order to construct this database, we recorded the skeletal information of these point-dances using a Kinect v2 sensor. The point-dances included in the K-pop dance database were composed of movements lasting for 4–9 s, and there were 25 skeletal joints considered. Among these joints, we selected 13 to obtain six core angles. The longest and shortest dance movements captured contained 147 and 276 frames, respectively. As mentioned in the previous section, we used a zero-padding method to produce frames of the same size. Zero padding padded the concatenated vector with zeros on both sides. Thus, the size of a point dance motion resultant vector was 6 × 276 elements. In this paper, we perform two different experiments. In the first experiment, the 800 total dance movements were divided into training and test sets of 400 movements each (one man and one woman). The total size of the training data set was 400 × 1656 elements. Here we used the data sequences showing the best results. In the second experiment, we performed 4-fold cross validation to test if the algorithm was independent from the dancer. Here we obtained the average rate of four classification results. Furthermore, we also performed the experiments regarding the normalized coordinates of shoulder, elbow, and knee joints. Figure 5 shows the environment of database construction using a Kinect camera. Figure 6 illustrates three examples of dance movements with sequential images.

### 4.2. Experiments and Results

**Figure 7:**
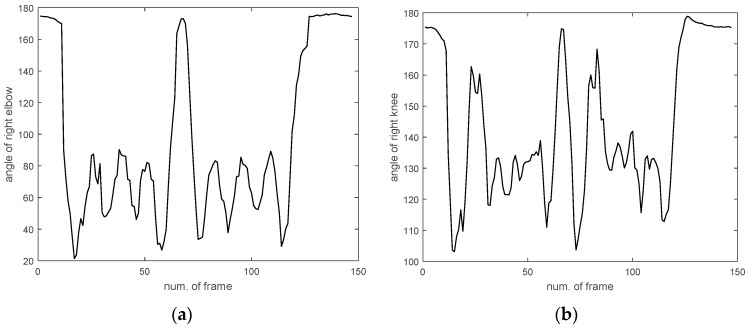
Right elbow and right knee angles (**a**) right elbow; (**b**) right knee.

**Figure 8:**
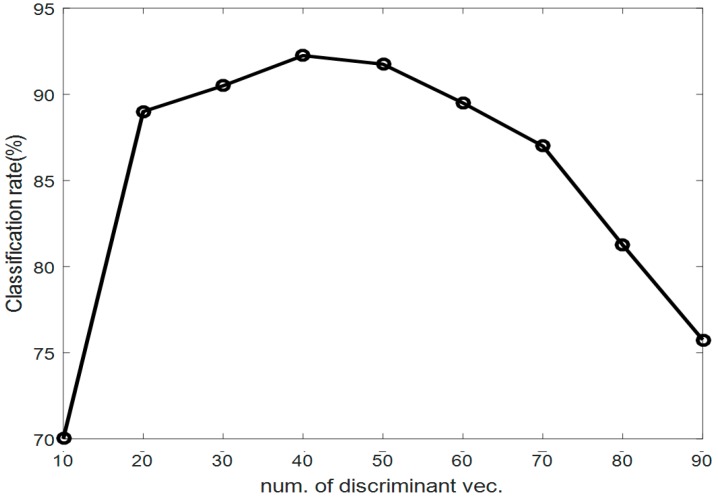
Classification rates based on PCA (Principal Component Analysis) + LDA (Linear Discriminant Analysis) (Euclidean distance).

In the first experiment, we compared the proposed method with conventional methods, such as the uses of KNN, SVM, and ELM alone. Figure 7 shows the right elbow and right knee angles, which were among the six angles representing a point-dance movement in each frame. After obtaining the concatenated vector, we selected *r* eigenvectors referring to the maximal recognition rate produced by the PCA method. Next, we determined the numbers of discriminant vectors *m* as the number of features in the LDA method increased. As a result, we selected the 100 eigenvectors that corresponded to the maximum recognition rate. From the obtained eigenvectors, we were able to determine that the use of 40 discriminant vectors provided the maximum recognition rate, as shown in Figure 8. 

**Figure 9:**
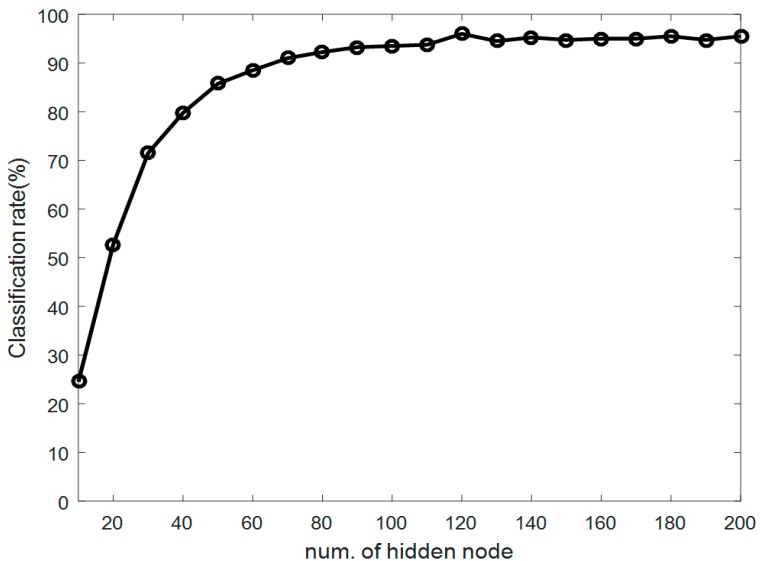
Classification rate according to the number of hidden nodes in the design of the ELMC.

**Figure 10:**
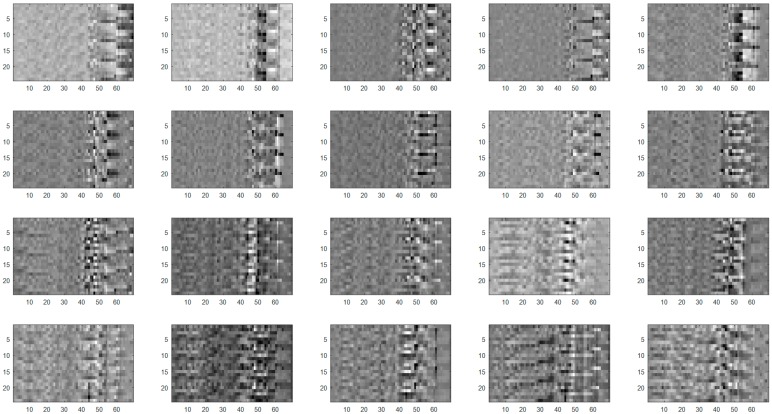
Fisherdance images.

Figure 9 shows the variation in classification rates as the number of hidden nodes in the ReLU-based ELMC design increases after the fisherdance method had been performed. We obtained a maximum classification rate of 96.5% when there were 120 hidden nodes. Table 1 compares the classification performance results of both the proposed method and the conventional methods. As listed in Table 1, the proposed method generally led to better classification results than the KNN, SVM, and ELM methods alone. Noticeably, the conventional ELM showed a worse performance than those of the conventional machine learning methods. Figure 10 shows fisherdance images representing the discriminant vectors defined in Equation (9). Here we visualize 20 discriminant vectors with the size of 1650 × 20. Each discriminant vector is converted into an image with a 24 × 69-pixel array with gray levels ranging from 0 to 255.

**Figure 11:**
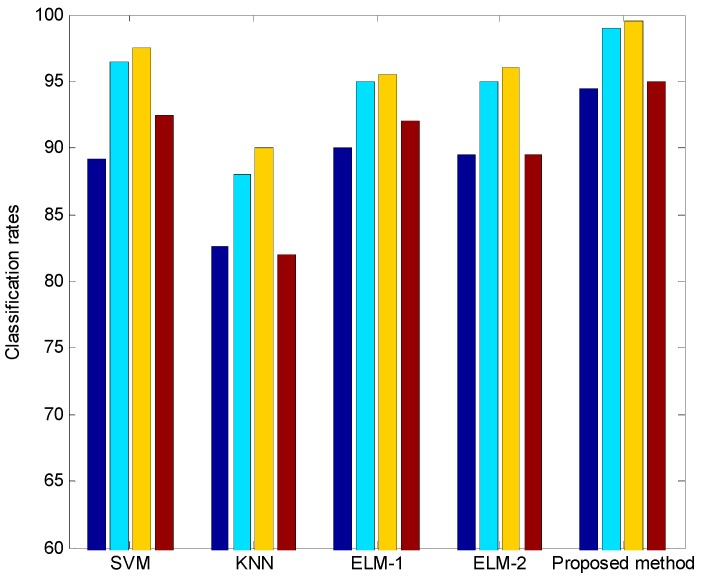
Each classification rate obtained by 4-fold cross validation.

In the second experiment, we performed 4-fold cross validation to test if the proposed method is independent from the dancer. That is, we used four data sets with 200 dance movements constructed by each professional dancer. Here, we also performed the experiments regarding the normalized coordinates of shoulder, elbow, and knee joints. Figure 11 visualizes the classification rates obtained by 4-fold cross validation. Table 2 lists the average rate of four classification results for the 4-fold cross validation method. As shown in Figure 11 and Table 2, it was found from the results that the proposed method showed a good performance in comparison with the SVM, KNN, and ELM methods with sigmoid and hard limit activation function. Table 3 lists the average classification rates for the 4-fold cross validation method with normalized coordinates. The results indicated that the normalization method in this study did not show a good performance in comparison with the general method without normalization.

## 5. Conclusions

We performed a point-dance movement classification via a combination of the fisherdance method and the ReLU-based ELMC. Furthermore, we constructed the first K-pop dance database with a total of 800 dance movements including 200 dance types obtained from four professional dancers by a Kinect sensor. The experimental results revealed that the proposed approach demonstrated a good performance in comparison with those of the methods used in previous works, including KNN, SVM, and ELM alone. Experimental results confirmed that the feature extraction of the concatenated vectors, the dimensional reduction performed by fisherdance, and the design of the proposed classifier were able to classify point-dance movements successfully. These results led us to the conclusion that the proposed method can be used effectively for various applications, such as dance plagiarism identification, dance training systems, and dance retrieval. In future research, we will analyze different sequential dance motions using DTW (Dynamic Time Warping) to solve the limitation of the fixed length of the feature vector. Furthermore, we will design a dance-movement classification system by integrating skeletal motion data with depth image sequences based on both a large dance movement database and deep learning.

## Figures and Tables

**Table 1:** Comparison of classification performance results.

Method	Dimensionality Reduction	Classification Rate (%)
KNN	—	77.75
	PCA + LDA	92.25
SVM	—	84.50
	PCA + LDA	92.75
ELM-1 (sigmoid)	—	43.00
	PCA + LDA	84.25
Proposed method	—	71.00
	PCA + LDA	96.50

**Table 2:** Comparison of the classification performance results for 4-fold cross validation.

Method	Dimensionality Reduction	Classification Rate (%)
KNN	—	53.81
	PCA + LDA	85.66
SVM	—	87.00
	PCA + LDA	93.92
ELM-1 (sigmoid)	—	50.37
	PCA + LDA	93.12
ELM-2 (hard-limit)		50.99
	PCA + LDA	92.5
Proposed method	—	77.61
	PCA + LDA	97.00

**Table 3:** Comparison of the classification performance results for 4-fold cross validation (normalization).

Method	Dimensionality Reduction	Classification Rate (%)
KNN	—	88.12
	PCA + LDA	92.50
SVM	—	62.75
	PCA + LDA	84.37
ELM-1 (sigmoid)	—	49.88
	PCA + LDA	91.12
ELM-2 (hard-limit)		48.63
	PCA + LDA	90.75
ReLU-based ELMC	—	75.49
	PCA + LDA	95.62
